# Malleable Penile Prosthesis Fractures: Lessons Learned from 17 Years of Experience at a Tertiary Center in Brazil

**DOI:** 10.1590/S1677-5538.IBJU.2026.0166

**Published:** 2026-06-20

**Authors:** Manuela Horta, Thiago A. Teixeira, Danilo C. Nagato, Carlos V. Neto, Gabriel P. O. Lima, Eduardo Z. S. Pato, Plinio M. Goes, José de Bessa, William C. Nahas, Jorge Hallak, Bruno C. G. Nascimento

**Affiliations:** 1 Faculdade de Medicina da Universidade de São Paulo Hospital das Clínicas Departamento de Cirurgia São Paulo SP Brasil Serviço de Urologia do Departamento de Cirurgia do Hospital das Clínicas da Faculdade de Medicina da Universidade de São Paulo - FMUSP, São Paulo, SP, Brasil; 2 Universidade Federal do Amapá Faculdade de Medicina Departamento de Cirurgia Macapá AP Brasil Divisão de Urologia, Departamento de Cirurgia, Faculdade de Medicina, Universidade Federal do Amapá - UFAM, Macapá, AP, Brasil; 3 Universidade de São Paulo Faculdade de Medicina São Paulo SP Brasil Faculdade de Medicina, Universidade de São Paulo, São Paulo – USP, São Paulo, SP, Brasil; 4 Universidade Estadual de Feira de Santana Faculdade de Medicina Departamento de Cirurgia Feira de Santana BA Brasil Divisão de Urologia, Departamento de Cirurgia, Faculdade de Medicina, Universidade Estadual de Feira de Santana – UEFS, Feira de Santana, BA, Brasil; 5 Faculdade de Medicina da Universidade de São Paulo Departamento de Patologia Unidade de Toxicologia Reprodutiva São Paulo SP Brasil Departamento de Patologia, Unidade de Toxicologia Reprodutiva, Faculdade de Medicina da Universidade de São Paulo - FMUSP, São Paulo, SP, Brasil; 6 Universidade de São Paulo Instituto de Estudos Avançados Grupo de Estudos em Saúde do Homem São Paulo SP Brasil Grupo de Estudos em Saúde do Homem, Instituto de Estudos Avançados (IEA-USP), Universidade de São Paulo, São Paulo, SP, Brasil

**Keywords:** Penile Prosthesis, Surgical Procedures, Operative, Comorbidity

## Abstract

**Purpose:**

To critically evaluate the clinical presentation, imaging performance and surgical findings of malleable penile prosthesis (MPP) fractures in a high-volume tertiary center.

**Materials and Methods:**

Medical reports of men who underwent revision surgery with intraoperative confirmation of MPP fracture between January 2008 and January 2025 were reviewed. MPP from a single manufacturer (Promedon^®^, Cordoba, Argentina) were inserted, and no comparisons were possible. Demographic data, presenting symptoms, imaging findings, and fracture location were analyzed. Diagnostic performance of physical examination and imaging modalities was descriptively compared.

**Results:**

Among 741 penile prosthetic procedures, 98 were revisions and 52 (53.1%) were due to MPP fracture. Median time from implantation to fracture was 59 months (IQR 24.8–84.0). Penile instability was the most common symptom (96.1%), while pain was reported in 21.1%. Physical examination correctly identified fractures in 88.5% of cases, outperforming radiography and magnetic resonance imaging. Bilateral fractures occurred in 51.9% of revisions, most commonly in the proximal segment. Recurrent fractures occurred in 28.2% of patients.

**Conclusions:**

MPP fractures are more prevalent than expected and a clinically relevant complication in high-volume centers. Diagnosis relies primarily on clinical assessment, with physical examination outperforming imaging modalities. Increased awareness of typical presentation patterns may support earlier recognition and more efficient management. Prospective studies are needed to identify modifiable risk factors, improve device design and quality, and advise patients on use to potentially improve prosthesis lifetime.

## INTRODUCTION

Erectile dysfunction (ED) affects over 50% of patients over the age of 40 (1), with prevalence increasing with aging, and a significant negative impact on both patients’ and their partners quality of life (QoL) (2). Despite the widespread use of phosphodiesterase type 5 inhibitors (PDE5Is) (3) and other effective treatment strategies such as shock wave therapy (4) or intracavernosal injections (5), penile prosthesis (PP) implantation remains a well-established treatment option, with high satisfaction rates among both patients and partners, and is considered the gold standard in those who fail, reject, or have contraindications to less invasive therapies (6).

Since its first description in 1936 (7), advances in prosthetic design have improved durability, concealability and rigidity (8), leading to the development of inflatable penile prosthesis (IPP) (9), which has yielded higher satisfaction rates among patients and their partners than the malleable penile prosthesis (MPP) (10). Recent advances have enabled penile prosthesis implantation to be safely performed with concomitant non-reconstructive urologic procedures, without increasing adverse events (11).

Nevertheless, MPPs offer important advantages over inflatable devices, including a more straightforward surgical technique, a lower incidence of mechanical complications, lower cost, and broader availability in developing countries (8, 12, 13). In Brazil, IPPs are neither covered by private insurance nor by the public Brazilian Universal Health Care System (Sistema Unico de Saude [SUS]), whereas MPPs are covered by both systems at no additional cost to patients. Similar access limitations to IPPs have been reported in other countries (14).

Although MPPs are associated with lower overall revision rates than IPPs (15), they are not without complications, including mispositioning (e.g., crossover), infection, extrusion, and fracture, all of which may ultimately lead to revision surgeries (8, 11, 14). Among these, MPP fractures remain poorly reported in the literature, with evidence largely limited to case reports, despite being relatively frequent in high-volume centers in developing settings.

We hypothesized that malleable penile prosthesis (MPP) fractures present with consistent clinical features that may facilitate early diagnosis and management, while fostering the comprehension of this event and promoting strategies for its prevention. Therefore, this study aims to describe the characteristics of surgically confirmed MPP fractures treated at a tertiary university hospital over a 17-year period.

## MATERIALS AND METHODS

After institutional ethical approval (CAAE-26509), we conducted a single-center cross-sectional study based on retrospectively collected data from medical records of adult men who underwent revision surgery for MPP fracture between January 2008 and January 2025. Only cases with intraoperative confirmation of implant fracture were included. Patients with intact MPPs intraoperatively or incomplete medical records were excluded. The study design is depicted in Figure-1. In Brazil, the law requires companies to submit proposals in a blind auction to sell their devices to the State Government; the company with the lowest price and that meets the quality and legal requirements is declared the winner. At Hospital das Clinicas, University of São Paulo Medical School, primary MPP implantations were performed using a standardized penoscrotal approach by Urology residents under the supervision of an experienced prosthetic surgeon. Almost all patients received the Tube Malleable Implant (Promedon^®^, Cordoba, Argentina) one patient had been previously reviewed, and one patient had previously received a Medicone implant (Medicone^®^, Porto Alegre, Brazil) at an outside institution. All revision surgeries at our center involved replacement with the Tube Malleable Implant (Promedon^®^, Cordoba, Argentina), the only device routinely available in our service.

**Figure 1:**
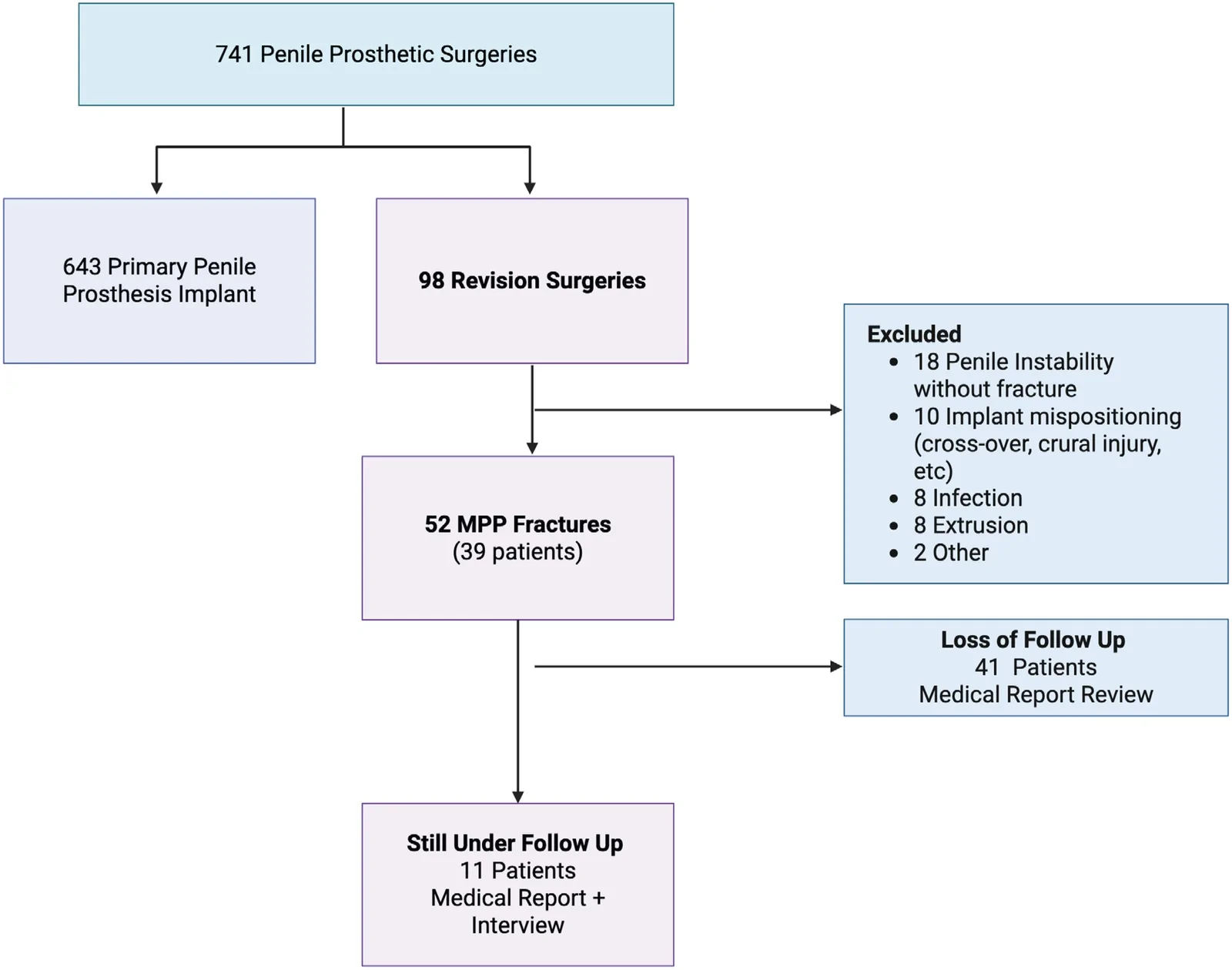
Flowchart of patient selection and study cohort.

Flowchart illustrating patient selection and study design. A total of 741 penile prosthetic surgeries were performed during the study period, including 643 primary implantations and 98 revision procedures. Among revision surgeries, 52 cases were due to malleable penile prosthesis (MPP) fracture and were included in the study. Revision surgeries for penile instability without fracture, implant mispositioning, infection, and extrusion were excluded. Of the 52 patients with confirmed MPP fracture, 41 were lost to follow-up, and 11 patients were available for detailed postoperative assessment and interview.

Routine postoperative follow-up was scheduled for two and six weeks, with clearance for sexual activity at the latter visit, followed by additional visits at three months and one year. Beyond this period, follow-up was patient-initiated as needed.

For patients with confirmed fracture, clinical and surgical data were collected, including age at implantation, erectile dysfunction etiology, comorbidities, prosthesis length and girth and post-operative complications.

Patients with confirmed fracture intraoperatively had clinical data of the implant procedure collected, including age at the time of implantation, etiology of erectile dysfunction (ED), comorbidities (eg, hypertension, diabetes, peripheral vascular disease, obesity, smoking history and chronic kidney disease), and intraoperative data, including prosthesis length and girth, the need for rear tip extensor utilization, and post-operative complications.

Fracture-related data were obtained from retrospective review of clinical records, including age at presentation, time from implantation to fracture and presenting symptoms such as pain, penile instability (inability to maintain axial rigidity or alignment during sexual activity) and impairment of penetrative intercourse.

Imaging studies were requested at the discretion of the attending physician without a standardized imaging protocol and included pelvic X-ray and/or magnetic resonance imaging (MRI). Available images were reviewed and compared to intraoperative findings to assess diagnostic accuracy.

From March 2023 onward, a standardized surgical report was implemented for suspected fracture cases, documenting the number and location of fractures and evidence of contralateral metallic core fatigue in addition to prosthesis length, girth and use of extender tips.

Sexual behavior data were not routinely collected before June 2023. Thereafter, information on sexual and masturbation frequency, preferred sexual position, anal penetration practice, use of lubricants and resting position of the PP was obtained during scheduled visits or structured telephone interviews.

Data was analyzed descriptively. Continuous variables are presented as median and interquartile range (IQR), while categorical variables are presented as frequencies and percentages. Statistical analyses were performed using GraphPad Prism, version 10.0.0 (GraphPad Software, Boston, MA).

## RESULTS

Between January 2008 and January 2025, 741 penile prosthetic surgeries were performed at our institution, including 98 revisions. Malleable penile prosthesis (MPP) fractures were the leading indication for reoperation (53.1%; 52/98), followed by penile instability without fracture (18.4%; 18/98), implant mispositioning (10.2%; 10/98), and infection or extrusion (8.2%; 8/98).

Fifty-two revision surgeries for MPP fracture were performed in 39 patients. Recurrent fractures were observed in 11 patients: 9 (23.1%) had two events whereas 2 (5.1%) had three separate events.

Median age at initial MPP implantation was 60.5 years (IQR, 54.5–66.2). Underlying etiologies of erectile dysfunction included metabolic syndrome (43.5%; 17/39), oncological conditions (38.5%; 15/39), Peyronie's disease (10.5%; 4/39), and spinal cord injury (7.5%; 3/39). Hypertension (74.4%; 29/39) and diabetes (35.9%; 14/39) were the most frequent comorbidities; with all but 1 patient having HbA1c < 8.5%. Additional comorbidities included smoking (17.9%; 7/39), chronic kidney disease or kidney transplantation (15.4%; 6/39), peripheral vascular disease (7.7%; 3/39), and obesity (BMI > 30; 5.1%; 2/39). No intraoperative complications were reported, and two patients developed early superficial wound dehiscence without need for surgical intervention.

Symptoms of MPP fractures developed a median of 59 months (IQR, 24.8–84.0) after implantation. Penile instability was reported by 96.1% of patients (50/52), whereas pain by 21.1% (11/52) and penetrative difficulty by 79.6% (40/52). Complete impossibility of penetration was reported by 15.4% (8/52), whereas 7.7% (4/52) reported no functional impairment.

Physical examination identified a palpable fracture in 88.5% (46/52) of cases. The evaluation consisted of systematic palpation of the penile shaft, combined with gentle axial compression and controlled bending of the prosthesis to assess discontinuity, abnormal angulation, or crepitus. Findings suggestive of fracture included a focal loss of rigidity, a palpable step-off, or crepitus along the shaft. Among patients who underwent X-ray, fracture was detected in 81.2% (26/32). Magnetic resonance imaging (MRI) was performed in 11 patients, but only 18.2% (2) of radiology reports described fractures. Representative imaging findings, including false-negative and discordant cases, are shown in Figure-2. Patient characteristics and diagnostic findings are summarized in Table-1.

**Figure 2:**
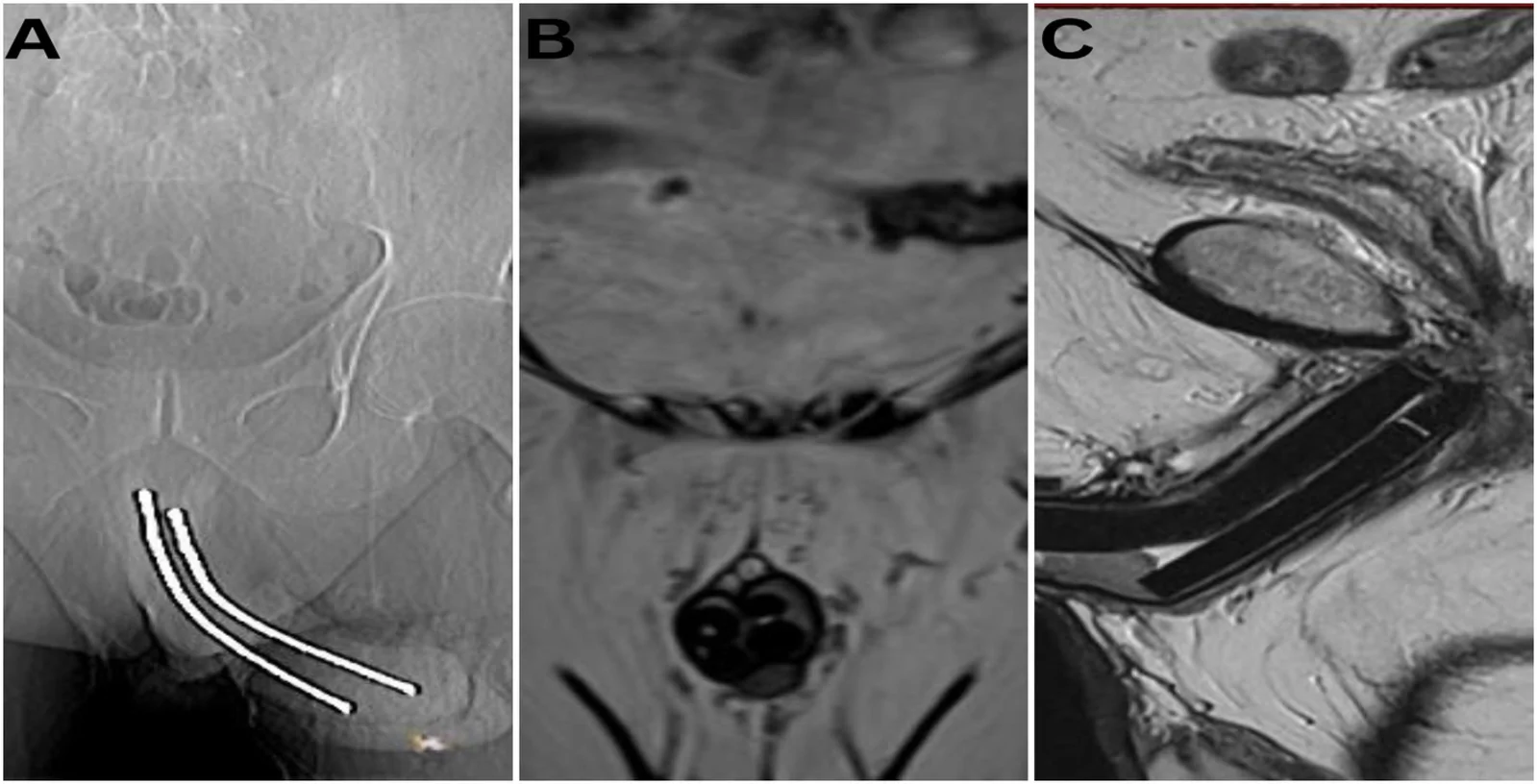
Imaging findings in malleable penile prosthesis (MPP) fracture (personal archive) highlighting discordance between imaging interpretation and intraoperative diagnosis.

(A) Plain radiograph of the patient illustrated in Figure-3A, with surgically confirmed bilateral MPP fracture, showing no evident abnormalities. (B) Magnetic resonance imaging (MRI) of the same patient illustrated in Figure-3A, also without clear evidence of prosthesis fracture. (C) MRI of a different patient initially reported as disconnection of rear tip extenders; however, intraoperative findings confirmed prosthesis fracture, as illustrated in Figure-3B.A

**Figure 3:**
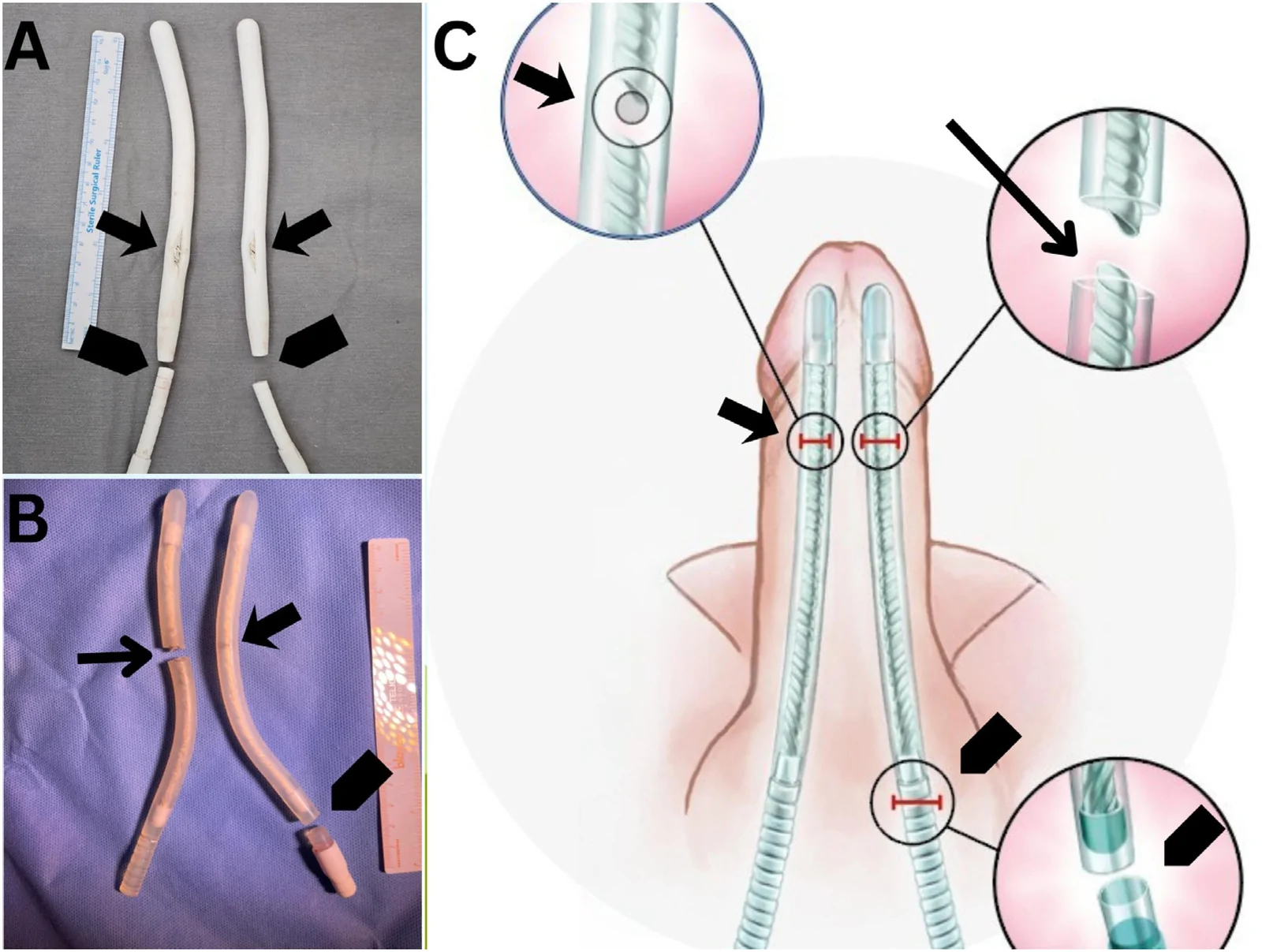
Intraoperative findings and fracture patterns of malleable penile prosthesis (MPP) (personal archive).

(A) Medicone prosthesis demonstrating multiple bilateral fractures. (B) Promedon prosthesis demonstrating multiple bilateral fractures. (C) Schematic representation of fracture patterns identified intraoperatively. Across all panels, thick arrows indicate metallic core fatigue without disruption of the silicone covering; arrowheads indicate complete fractures external to the metallic core; and thin arrows indicate complete fractures involving both the silicone covering and the metallic core.

**Table 1:** Signs and symptoms reported by patients with malleable penile prosthesis (MPP) fracture.

Variable	Value
Total Sample - MPP Fractures	(n = 52)
Time from MPP Implant to MPP Fracture Complaint	
< 1 year	11.5% (n = 6)
1 - 5 years	40.4% (n = 21)
5 - 10 years	40.4% (n = 21)
> 10 years	7.7% (n = 4)
MPP Fracture Symptoms	
Instability	96.1% (n = 50)
Pain	21.1% (n = 11)
Challenge with Intercourse Penetration	
Difficulty with penetration	76.9% (n = 40)
Impossibility of penetration	15,4% (n = 8)
Penetration not impaired	7.7% (n = 4)
MPP Physical Exam	
Abnormal	88.5% (n = 46)
Normal	11.5% (n = 6)

Data are presented as n (%); MPP = malleable penile prosthesis; IQR = interquartile range

All revision procedures were completed without intraoperative complications. Bilateral fractures were present in 51.9% (27/52). In 53.8% (28/48) of revisions, the replacement implant had greater girth and/or length. Fracture distribution by girth was: 9 mm (19.2%; 10/52), 10 mm (43.2%; 22/52), and 11 mm (21.1%; 11/52). No fractures occurred in 12 - 13mm prosthesis, which were unavailable before 2019. Median prosthesis length was 20cm (IQR 19-21.5). Rear tip extenders were used in only 15.4% (8/52) of cases. Sizing was primarily based on girth.

Forty-four surgical reports were available for detailed analysis (Figure-3), which illustrates representative intraoperative findings and fracture patterns. Up to four wear points per prosthesis were identified; and multiple wear points per cylinder were observed in 61.3% (27/44). Fractures were predominantly proximal (77.1%; 27/35), followed by distal (22.6%; 8/35) and mid-cylinder (17.1%; 6/35). Sexual behavior data were available for 11 patients. Most reported a single partner (72.7%; 8/11). None reported anal penetration. Lubricant use was routine in 55.0% (6/11). All patients routinely positioned the penis laterally for concealment. Median monthly penetrative intercourse before fracture was 2.0 (IQR 1.2–3.8), with a maximum of 12; two patients reported no intercourse. Among the 11 patients, the most common position was the man in the superior position over the partner (72.7%; 8/11), followed by the same position with the partner's leg elevated (36.4%; 4/11). Less frequent positions included partner-on-top (9.1%; 1/11) and kneeling male with the partner supine and pelvis elevated (9.1%; 1/11).

## DISCUSSION

Penile prosthesis (PP) implantation is a well-established therapeutic option for erectile dysfunction (ED) and Peyronie's disease (14). Because implantation is irreversible, understanding complication patterns and failure mechanisms is paramount for patient counseling and long-term satisfaction.

In the United States, inflatable penile prosthesis (IPP) account for approximately 90% of implants, largely due to insurance coverage and higher patient and partner satisfaction (7, 8, 10). In contrast, malleable penile prosthesis (MPP) remains widely used in developing countries owing to lower cost and easier of installation and functionality, demanding less steps for satisfactory sexual intercourse, but more prone to repeated bending (14). As a result, most available data derives from IPP-dominant populations, rendering MPP-related complications comparatively underexplored.

Large population-based studies suggest comparable overall revision rates between both prosthesis types. Lacy et al. (15) compared revision surgery rates among 6,586 patients undergoing primary penile prosthesis implantation with at least one year of follow-up, of whom 13.4% (883) received malleable penile prostheses (MPP). Their analysis demonstrated longer time to revision for MPP compared with IPP at 1-, 5-, and 10-year follow-up intervals (HR: 0.68; 95% CI 0.53–0.83; p < .001), with a statistically significant divergence after 13 years, suggesting favorable long-term durability of malleable devices. Similarly, Grewal et al. (12) analyzed 2,263 penile prosthesis implantations in California between 2006 and 2009, of which 19.4% (439) were MPP, and found no significant difference in overall reoperation rates (7.52% MPP vs 7.40% IPP; p = 0.94), although complication patterns differed, with a higher proportion of infectious complications observed after MPP implantation (4.5% MPP vs 3.23% IPP; p = 0.18) and a greater frequency of noninfectious complications following IPP placement (2.96% MPP vs 4.17% IPP; p = 0.25). Importantly, neither study reported prosthesis manufacturer nor stratified early and late complications, limiting device-level interpretation.

Evidence regarding MPP-specific complications, particularly fracture, remains scarce and largely limited to case reports or secondary observations in studies designed for other outcomes. Despite this limited evidence base, our series demonstrates that MPP fracture was the leading cause for revision in our institution, accounting for 53.1% of reoperations. Our results, however, should be interpreted within the context of the study design. As only patients undergoing revision surgery with intraoperatively confirmed fractures were included, our findings reflect the relative contribution of fractures among revision cases in our institution and do not allow estimation of true incidence, prevalence or population-level occurrence.

In 2006, Minervini et al. (16) reported two cases of MPP rod fracture, one in the former model, the AMS 600 (Boston Scientific, Marlborough, MA) after 112 months and one Montor Malleable (Montor Medical, São Paulo, Brazil) after 79 months. Mohamed et al. (17) reported three revision surgeries due to MPP fractures in Promedon Tube Prosthesis after 13, 19, and 22 months of their implantation, being the cause of 23% (3/13) of prosthesis removal and 8.3% (3/36) of long-term complications in this cohort. Moreover, Pinheiro et al. (18) reported multiple site fractures on both cylinders of a 72-year-old patient, being the first to describe multiple fractures per cylinder.

Within the Brazilian Public Unified Health Care System (Sistema Unico de Saude [SUS]), device selection is determined by a public bidding process prioritizing cost-effectiveness once predefined technical criteria are met. Consequently, the Promedon Tube Malleable Implant (Promedon^®^, Cordoba, Argentina) has been the primary device at our center. This prosthesis consists of a silicone elastomer body with a polytetrafluoroethylene–coated twisted silver wire core designed to provide axial rigidity (19, 20). A similar internal architecture is observed in the Medicone Penile Implant (Medicone^®^, Porto Alegre, Brazil) identified in one of our revision surgeries. Notably, both designs resemble earlier-generation MPP designs, such as the one by Dr Udo Jonas, introduced in the US marketplace in 1980 but soon replaced as the silver strands tended to fracture, rendering device failure (8).

Behavioral factors have been proposed as potential contributors to MPP fracture risk. Prior studies suggest that vigorous sexual activity, particularly positions where the woman is on top, may increase axial load (16, 17). Anal penetration has also been hypothesized as a contributing factor - according to Miller et al. (21), anal penetration requires a mean axial force of 2700 g (2640–4240 g), exceeding the approximately 1500 g required for vaginal penetration reported by Karacan (22). However, in our cohort, behavioral data were limited and did not allow confirmation or refutation of these proposed risk factors. Moreover, the small sample size (n = 11) precludes any meaningful conclusions, and these findings should be interpreted as exploratory and hypothesis-generating, granting future data collection for clarification.

Beyond behavioral aspects, engineering and materials science principles are central to understanding device failure. From a biomechanical perspective, ED can be conceptualized as collapse or buckling of the penile column under an axial load during penetration (23). Manufacturers report varying axial load resistance thresholds across MPP models. The Promedon Tube Malleable Implant (Promedon^®^, Cordoba, Argentina) is designed to withstand axial loads exceeding 1,500 grams without bending (19), whereas the Tactra™ (Boston Scientific Corporation, Marlborough, MA) was engineered to provide a column strength from a pair of cylinders of 2,767 grams (24). Al Ansari et al. (25) utilized a digital inflection rigidometer (DIR) to assess the penile rigidity finding a mean DIR of 857g for a pair of cylinders placed in patients of the Genesis^®^ MPP (Coloplast Corp., Minneapolis, MN). These thresholds generally permit penetrative intercourse under physiologic conditions, but do not fully account for long-term mechanical fatigue.

Fracture susceptibility, however, depends not only on peak axial resistance, but also on geometry and material fatigue. Differences in device girth may alter resistance to bending forces, analogous to greater fragility of thinner rods compared to thicker ones of the same material (26; 27;28). In our series, no fractures were observed in 12 or 13mm rods; however, this finding should be interpreted with caution, as these devices had shorter follow-up, frequently below the median time to fracture in our cohort. While increased mechanical strength with larger diameters may be hypothesized, our design does not allow a robust comparison, as patients without fracture were not systematically analyzed.

More importantly, repetitive bending during concealment, voiding, or repositioning, exerts chronic stress particularly at the proximal shaft. In our cohort 77.1% occurred in this site, supporting cumulative fatigue as the predominant failure mechanism rather than acute overload. While the role of rear tip extenders (RTEs) in reducing axial rigidity and contributing to mechanical stress has been described in inflatable prostheses, these findings cannot be directly extrapolated to malleable devices. Unlike inflatable prostheses, malleable implants have a more homogeneous structure, and the difference in mechanical properties between the prosthesis and the extender is less pronounced. Taken together, these findings support a conceptual interpretation of failure mechanisms integrating cyclic fatigue, implant geometry, potential behavioral and manipulation (bending frequently) factors, grounded in both our data and established biomechanical principles. Future prospective studies with standardized reporting are warranted to further refine and validate these models.

Although a formal classification of fracture types was not feasible in our cohort due to limitations in intraoperative documentation, our findings support a conceptual model in which cyclic fatigue, implant geometry, and mechanical stress distribution contribute to device failure. The predominance of proximal fractures suggests a fatigue-related mechanism rather than acute overload.

These findings should be interpreted in the context of important methodological limitations. Imaging studies were non-standardized, performed at physician discretion, and not reviewed in a blinded fashion, limiting direct comparison of diagnostic performance across modalities. Proximal silicone fractures outside the metal axis were radiolucent on X-rays (Figure-2A). The low diagnostic yield of MRI observed in our cohort is unlikely to be explained by artifact-related blooming, as this effect is not prominent with the materials used in contemporary MPPs. Instead, the limited accuracy of MRI in this setting may reflect intrinsic methodological challenges, including difficulty in clearly delineating prosthesis discontinuity due to component overlap and subtle structural changes. In addition, limited familiarity of radiologists with prosthesis-specific failure patterns may further contribute to under-recognition. MRI interpretation was frequently limited by component overlaps without clear fracture visualization (Figure-2B).

Currently, no comparative studies systematically evaluated axial load tolerance and fatigue resistance across commercially available MPP brands (15). However, these results should be interpreted in the light of the fact that our cohort included a single device type (Promedon Tube Malleable Implant), characterized by a twisted silver wire core. Therefore, our findings may not be fully generalized to other MPP architectures such as Nitinol-based or helical-core designs, which may exhibit different mechanical properties and fatigue resistance profiles. These differences in internal architecture may influence long-term durability and fracturing patterns.

This study has limitations inherent to its retrospective, single-center design and absence of standardized long-term follow-up. As only patients undergoing revision surgery with confirmed fractures were included, the study is subject to selection bias and does not allow estimation of true incidence and prevalence. Routine annual follow-up is uncommon in prosthetic urology, limiting the ability to identify modifiable risk factors. Additionally, results primarily reflect experience with a single MPP model.

Nevertheless, this study represents, to our knowledge, the largest reported series of surgically confirmed MPP fractures, providing a comprehensive analysis of clinical presentation, diagnostic performance, and intraoperative findings over 17 years of experience and may provide guidance for future more resilient implants.

## CONCLUSIONS

Malleable penile prosthesis fractures are an underreported yet clinically relevant complication and represented the leading indication for revision surgery in our cohort. Diagnosis relies primarily on careful physical examination, which outperformed conventional imaging modalities and may be sufficient to justify surgical revision in selected cases.

Fractures were frequently bilateral and predominantly located in the proximal segment, supporting cumulative material fatigue as the primary failure mechanism. Clinicians should maintain a high index of suspicion in patients presenting with penile instability or impaired penetrative intercourse and counsel patients accordingly.

Standardized and transparent reporting of mechanical performance, including axial load tolerance and resistance to cyclic fatigue, is needed to better inform clinical decision-making and device selection. Prospective, multicenter studies comparing different malleable penile prosthesis models are warranted to define modifiable risk factors and improve long-term outcomes and guide future development of more resilient models.

## Data Availability

Trial data will be made available on reasonable request to the corresponding author.
